# A Radical Newspaper and British Workmen's Interests

**Published:** 1911-12-09

**Authors:** 


### A RADICAL NEWSPAPER AND BRITISH WORKMEN'S INTERESTS.

We hope the following instance of the way in which the *Daily Chronicle* treated the Conference of Hospital Managers, may become known to British workmen through the Hospital Saturday Funds and the Press. The *Daily Chronicle* once gloried in being a great newspaper, impartial, fair and just to all the interests it represented. The Conference of Hospital Managers merely represented matters of vital importance to the bread-winners of the United Kingdom, which our contemporary apparently holds to be of no importance, compared with the supposed interests of a political party. The *Daily Chronicle's* only reference to the British Hospitals Conference was the following:—

HOSPITAL SCARE EXPLODED.

Another Insurance Bill scare was raised at a conference of hospital representatives yesterday at the Westminster Palace Hotel, London.

Sir Hy. Burdett declared that insured persons were ineligible for hospital treatment, and wanted this alleged fact sent out broadcast to the friendly societies, etc. As proof of his statements he said that some months ago Mr. Lloyd George stated it would be open to the governors to ask any insured patient in a hospital, "Why are you here?"

It is evident, however, that he misunderstood the Chancellor, or that the Chancellor thought that in most cases other than hospital treatment will be provided.

Certainly it is untrue to suggest that working men will be deprived of hospital treatment, for Clause 14 makes special provision for "the case of contributors who are inmates of hospitals, etc." This clause prevents the sick benefit being seized by the hospital authorities, and says it shall be paid for the maintenance of the patient's dependents. If, however, he has no dependents, it may be paid to the hospital if the society of which he is a member has an agreement to that effect.

One may point out, too, that Clause 20 empowers the approved societies to make subscriptions, donations, etc., to hospitals, obviously in recognition of the benefits their members will receive.

Sir Henry Burdett immediately replied:—

"HOSPITAL SCARE EXPLODED."

Sir,—The paragraph under the above heading in the *Daily Chronicle* of this date is not worthy of the best traditions of a great newspaper. Both Canon Henson and myself showed that Clause 14 only applied to the merest fraction of the total number of insured persons, and that it would be practically inoperative, because it required in every case an agreement for the purpose involved and a claim to some control in the management of the hospital which, under the most favourable conditions, could not receive more than about a third of the cost incurred by the institution for such sick patients.

Clause 20 was also shown to be no provision for or of hospital treatment for the insured. For in the case of London alone 5,000 hospital beds will be required for the sole use of insured persons, the cost of maintaining which represents a sum of from £400,000 to £500,000 a year. The subscriptions and donations mentioned in this clause could never, therefore, be anything but a small percentage of this sum, which must be forthcoming, or the insured cannot receive "the adequate medical treatment" which Mr. Lloyd George said at Birmingham on June 12 "the Bill will provide for every workman in the United Kingdom." Further, on introducing the Bill Mr. Lloyd George claimed that it provided free medical relief with no taint of charity, whereas the provisions of both Clauses 14 and 20 involve charitable relief to the insured affected by them. Again in his speech to the deputation of Hospital Managers on July 29, Mr. Lloyd George said the hospitals should ask the patient, "Are you insured?" "If they were, then they had no right to come to the hospitals."

It is therefore the fact that your correspondent, though he has ignored these material facts which were stated at the meeting, has not only not exposed a "hospital" or any "scare," but will, I hope, with your help, enable the actual position of the insured in regard to hospital care to be widely known and appreciated.

The voluntary hospitals under their constitution provide free hospital treatment for those who, though able to maintain themselves and their families when well, cannot pay the cost of illness in their own case or that of their dependents. That is why insured persons will no longer be eligible to receive free in-patient treatment at a voluntary hospital, for they are to be provided with adequate medical treatment in virtue of their own payments and those of their employers and the State.

Even if they were eligible, where is the money to come from to defray the cost of such benefits? The voluntary hospitals, which are estimated to lose some 50 per cent. of their present revenue, cannot find it. If they could, the insured would have to accept charity whenever they entered a hospital. So, alas, Government will see, there is no "scare," but sober truth in the statement that the insured, as the Bill stands, will be ineligible for free hospital treatment.

A certain place is said to be lined with good intentions. But when it comes to be, as in this case, a question of the health and lives of the workmen of the United Kingdom, the promises of a British Chancellor of the Exchequer must be made good, all will agree, before the Insurance Bill becomes an Act of Parliament.

I am, Sir, yours faithfully,

(Sd.) Henry C. Burdett.

The Editor, *Daily Chronicle.*
December 2, 1911.

Instead of publishing this letter the *Daily Chronicle* disposed of the matter in its issue of 4th instant by the following paragraph:

"HOSPITAL SCARE EXPOSED."

In reference to the statement at the Hospital Managers' Conference in London (under the above heading) that insured persons would not have their former right to hospital treatment, and our reply that Clauses 14 and 20 of the Bill provide for payment to the hospitals for taking such patients, Sir Henry Burdett writes that

"Clause 14 will be practically inoperative, because it requires in every case an agreement for the purpose, and involve a claim to some control in the management of the hospital, which, under the most favourable conditions, could not receive more than about a third of the cost incurred by the institution for each patient."

With regard to Clause 20, which empowers approved societies to subscribe to hospitals, Sir Henry says:—

"It makes no provision for hospital treatment for the insured. For in the case of London alone 5,000 hospital beds will be required for the sole use of insured persons, the cost of maintaining which represents from £400,000 to £500,000 a year. The subscriptions and donations mentioned in this clause could never, therefore, be anything but a small percentage of this sum.

"The voluntary hospitals, under their constitutions, provide free hospital treatment for those who though able to maintain themselves and their families when well, cannot pay the cost of sickness in their own case or that of their dependents. That is why insured persons will no longer be eligible to receive free in-patient treatment at a voluntary hospital, for they are to be provided with adequate medical treatment in virtue of their own payments and those of their employers and the State."

